# Role of HTLV-1 Tax and HBZ in the Pathogenesis of HAM/TSP

**DOI:** 10.3389/fmicb.2017.02563

**Published:** 2017-12-21

**Authors:** Yoshimi Enose-Akahata, Ashley Vellucci, Steven Jacobson

**Affiliations:** Viral Immunology Section, Division of Neuroimmunology and Neurovirology, National Institute of Neurological Disorders and Stroke, National Institutes of Health, Bethesda, MD, United States

**Keywords:** HTLV-1, HAM/TSP, Tax, HBZ, neuroinflammatory disease

## Abstract

Human T cell lymphotropic virus type 1 (HTLV-1) infection can lead to development of adult T cell leukemia/lymphoma (ATL) or HTLV-1-associated myelopathy/tropical spastic paraparesis (HAM/TSP) in a subset of infected subjects. Understanding the interaction between host and HTLV-1 and the molecular mechanisms associated with disease pathogenesis is critical for development efficient therapies. Two HTLV-1 genes, *tax* and *HTLV-1 basic leucine zipper factor* (*HBZ*), have been demonstrated to play important roles in HTLV-1 infectivity and the growth and survival of leukemic cells. Increased HTLV-1 Tax expression induces the expression of various cellular genes such as IL-2 and IL-15, which directly contributes to lymphocyte activation and immunopathogenesis in HAM/TSP patients. However, little is known about the molecular and cellular mechanism of HBZ in development of HAM/TSP. It has been reported that *HBZ* mRNA expression was detected in HAM/TSP patients higher than in asymptomatic carriers and correlated with proviral load and disease severity. Unlike HTLV-1 tax, HBZ escapes efficient anti-viral immune responses and therefore these reactivities are difficult to detect. Thus, it is important to focus on understanding the function and the role of HTLV-1 tax and HBZ in disease development of HAM/TSP and discuss the potential use of these HTLV-1 viral gene products as biomarkers and therapeutic targets for HAM/TSP.

## Introduction

Human T lymphotropic virus type 1 (HTLV-1) is the first human retrovirus discovered belonging to the deltaretrovirus family and is thought to infect approximately 10–20 million people worldwide (Poiesz et al., 1980; de The and Bomford, 1993). Several highly endemic areas for HTLV-1 are known in the world such as southern part of Japan, the Caribbean, North and South America, Central and West Africa, and foci in Middle East, Australia and Melanesia (Gessain and Cassar, 2012). HTLV-1 has been demonstrated to be the etiological agent of an aggressive mature T cell malignancy termed adult T cell leukemia (ATL) (Uchiyama, 1988) and a chronic, progressive neurological disease termed HTLV-1-associated myelopathy/tropic spastic paraparesis (HAM/TSP) (Gessain et al., 1985; Osame et al., 1986). Although the majority of infected individuals are asymptomatic carriers of the virus, approximately 0.25–3.8% of infected individuals develop HAM/TSP. HAM/TSP is clinically characterized by progressive lower extremity weakness, spasticity, and bladder/bowel sphincter dysfunction (Umehara et al., 1993). The disease development of HAM/TSP mainly occurs in adults, with a mean age at onset of 40–50 years, which is more common in women than in men (Yamano and Sato, 2012). The disease usually progresses slowly without remission, but the clinical course and rate of progression may vary greatly among patients (Yamano and Sato, 2012). HAM/TSP is also characterized by perivascular inflammatory infiltrates in the brain and spinal cord (Umehara et al., 1993; Aye et al., 2000). In early stages of HAM/TSP, infiltrating CD4^+^ and CD8^+^ lymphocytes are present in the inflammatory lesions in the spinal cord while CD8^+^ T cells are predominantly detected in the chronic inflammatory lesions of patients with longer duration of disease (Umehara et al., 1993). The spinal cord is associated with a chronic inflammatory process with marked parenchymal exudation of inflammatory cells in both the gray and white matter, initially causing lower thoracic cord atrophy with extensive lateral and posterior column involvement (Iwasaki, 1990). As the disease progresses, axonal loss and degeneration in the form of myelin pallor is severe in the white matter and increases throughout the entire cord (Iwasaki, 1990). Recent studies of measuring spinal cord cross-sectional areas in HAM/TSP patients by magnetic resonance imaging (MRI) revealed that HAM/TSP patients had more atrophic cords compared to ACs and healthy normal donors, which was associated with disease duration (Liu et al., 2014; Azodi et al., 2017). In HAM/TSP patients who present with rapid progression, spinal cord atrophy has been demonstrated to begin in the thoracic cord and progress to the cervical cord (Azodi et al., 2017). A higher HTLV-1 proviral load (PVL) is frequently observed in the blood and the cells from cerebrospinal fluid (CSF) of HAM/TSP patients than ACs (Nagai et al., 2001b). High levels of antibodies against HTLV-1 antigens are present in blood and CSF (Gessain et al., 1985; Osame et al., 1986). Intrathecal HTLV-1-specific antibody production provides additional data to support the diagnosis of HAM/TSP. Thus, chronically activated immune responses against HTLV-1 and infiltration of inflammatory cells including HTLV-1 infected cells into the central nervous system (CNS) have been suggested to underlie the pathogenesis of HAM/TSP.

Like other retroviruses, the HTLV-1 proviral genome has structural genes, *gag, pol*, and *env* flanked by long terminal repeat at both ends. HTLV-1 genome also contains a *pX* region between *env* and 3′ LTR encoded several accessory genes including *tax, rex, p12, p21, p30, p13* and *HTLV-1 basic leucine zipper factor* (*HBZ*) (Matsuoka and Jeang, 2007). The viral genes are transcribed from the 5′ LTR, but only *HBZ* encoded on the minus strand of the provirus is transcribed from the 3′ LTR. HTLV-1 expresses a transcriptional *trans*-activator protein, Tax, which induce the expression of a various cellular genes. Increased expressions of critical immune mediators directly contribute to cell activation and proliferation observed in HAM/TSP patients, suggesting that chronically activated immune responses underlie the pathogenesis of this disorder (Matsuura et al., 2010). There is also increasing evidence that HBZ also plays a critical part in inflammation and pathogenesis of HAM/TSP. In this review, we discuss the understanding of the function and the role of HTLV-1 Tax and HBZ in disease development of HAM/TSP and the potential use of these HTLV-1 viral gene products as biomarkers and therapeutic targets for HAM/TSP.

## HTLV-1 Infection and HAM/TSP

HTLV-1 causes a persistent infection in humans and replicates mainly through clonal expansion of the infected cells rather than cell-free virus infection. HTLV-1 PVLs are significantly elevated in HAM/TSP patients, compared to ACs, and is strongly correlated with disease pathogenesis of HAM/TSP (Nagai et al., 1998). Higher PVLs are detected in CSF than in peripheral blood mononuclear cells (PBMCs) of HAM/TSP patients (Nagai et al., 2001b; Araya et al., 2014; Brunetto et al., 2014). In addition, PVL was significantly higher in CSF of HAM/TSP patients than in ACs and HTLV-1-infected subjects with other neurologic diseases (Puccioni-Sohler et al., 2007). HTLV-1 can infect a wide range of human cell types including T cells, monocytes, macrophages and dendritic cells (Hoffman et al., 1992; Koralnik et al., 1992; Koyanagi et al., 1993; Makino et al., 1999; Hanon et al., 2000c; Enose-Akahata et al., 2008). HTLV-1 provirus is predominantly detected in CD4^+^ T cells *in vivo* (Richardson et al., 1990) and has been associated with leukemogenesis and reduced regulatory function of CD4^+^ T cells (Uchiyama, 1988; Yamano et al., 2005). In HAM/TSP patients, CD4^+^CD25^+^ T cells are the main reservoir for HTLV-1 (Yamano et al., 2004). Further studies revealed that high HTLV-1 PVL was detected in CD4^+^CD25^+^CCR4^+^ T cells and the frequency of IFN-γ-producing CD4^+^CD25^+^CCR4^+^ T cells was dramatically increased in HAM/TSP patients, which was found to be correlated with disease activity and severity (Yamano et al., 2009; Araya et al., 2014). In addition, it has been demonstrated that abundant CD4^+^CCR4^+^ T cells which coexpressed the Th1 marker CXCR3 and produced T-bet and IFN-γ were present in CSF and spinal cord lesions of HAM/TSP patients (Araya et al., 2014). High CSF PVLs is a strong biomarker of HAM/TSP and HTLV-1-infected cells recruited into the CNS may alter the inflammatory milieu in the CNS of HAM/TSP patients.

HTLV-1 PVL varies widely among HTLV-1-infected subjects, but the PVL remains relatively stable within each subject. The HTLV-1 genome sequence is also stable within and between infected individuals. Integration of HTLV-1 was found to favor genes, transcriptional start sites, and CpG islands (Doi et al., 2005; Derse et al., 2007). Comparison of proviral integration sites between HTLV-1-infected subjects demonstrated that HTLV-1 integration might be more frequent in transcriptionally active areas of the genome in HAM/TSP patients than in ACs and that frequent integration into transcriptionally active area of the genome was associated with an increased rate of Tax expression (Meekings et al., 2008). Moreover, a larger number of unique insertion sites, but not a difference of clonality, was detected in HAM/TSP patients than in ACs (Gillet et al., 2011). Interestingly, the majority of spontaneous Tax expressing cells corresponded to the large number of low abundance clones, rather than a small number of high abundance clones (Melamed et al., 2013), suggesting that clonal expansion of infected cells might be controlled by host immune response to Tax or by other viral factor such as HBZ in HAM/TSP patients. These reports demonstrated that cells with HTLV-1 provirus integrated near transcriptionally active areas could establish and expand more frequently in HAM/TSP patients, which would influence expression of HTLV-1 gene products and further contribute to the development and pathogenesis of HAM/TSP.

## Molecular Pathogenesis of Tax in HAM/TSP

Tax is a transforming and transactivating protein of HTLV-1 and induces the expression of a variety of cellular genes by activation of the NF-κB and CREB/ATF pathways (Matsuoka and Jeang, 2011). HAM/TSP patients showed the spontaneous increase of *tax* mRNA and Tax protein expression in PBMCs after *ex vivo* culture without any exogenous stimulators that peaks at 12–24 h (Hanon et al., 2000a; Yamano et al., 2002). The expression of *tax* mRNA was significantly higher in HAM/TSP patients than in ACs (Yamano et al., 2002). Tax is prominently associated with dysregulation in immune cells of HAM/TSP patients, underlying many of the characteristic immune abnormalities.

### Regulatory CD4^+^ T Cells (Tregs)

In HAM/TSP patients, CD4^+^CD25^+^ T cells contain high frequency of HTLV-1 proviral DNA, express HTLV-1 *tax* mRNA at significantly higher levels than in CD4^+^CD25^-^ cells and induces various cytokines including IFN-γ (Yamano et al., 2004, 2009). CD4^+^CD25^+^ T cells, termed Tregs, that constitutively express CD25 (the IL-2 receptor α chain) and are engaged in the maintenance of immunologic self-tolerance by suppressing the activation and expansion of self-reactive lymphocytes that may cause autoimmune diseases (Sakaguchi et al., 2001). Although CD4^+^CD25^+^ T cells have an important role in suppression of T cell activation both *in vivo* and *in vitro*, HTLV-1-infected CD4^+^CD25^+^ T cells were not functionally suppressive but rather were shown to stimulate the proliferation of HTLV-1 Tax-specific CD8^+^ T cells (Yamano et al., 2004). In HAM/TSP patients, the forkhead box P3 (FoxP3), which is critical for the function of Tregs, was decreased in CD4^+^CD25^+^ T cells (Yamano et al., 2005). When Tax was transduced in CD4^+^CD25^+^ T cells isolated from healthy volunteers, *foxp3* mRNA expression as well as regulatory function of the CD4^+^CD25^+^ T cells was inhibited (Yamano et al., 2005).

Other immune molecules related to Tregs were also dysregulated by Tax in HAM/TSP patients. The pleiotropic cytokine, transforming growth factor-β (TGF-β), play critical roles in suppressing the immune response, such as inhibition of inflammatory responses and promotion of Treg generation and function (Wan and Flavell, 2007). Dysregulation of TGF-β signaling has been reported in HAM/TSP patients, due to inhibition of TGF-β RII and Smad7 expression by Tax (Grant et al., 2008). OX40 is a member of the TNF co-stimulatory receptor family and is expressed on activated T cells. Costimulatory signals from OX40 promote proliferation and survival of effector and memory T cell population and also suppresses the differentiation and activity of Tregs (Croft et al., 2009). Tax has been demonstrated to transactivate OX40 (Higashimura et al., 1996). In HAM/TSP patients, OX40 was expressed in CD4^+^ T cells depending on Tax expression after the culture (Saito et al., 2013). Higher levels of soluble OX40 was detected in the CSF of HAM/TSP patients with rapid progression, and OX40 was overexpressed in spinal cord infiltrating mononuclear cells in a clinically progressive HAM/TSP patient with a short duration of illness (Saito et al., 2013). Thus, decreased Treg function may cause immune dysregulation in HAM/TSP patients.

### The Common γ Chain Family of Cytokines

The common γ chain family of cytokines including IL-2, IL-7, IL-9, IL-15, and IL-21 play an important role in lymphocyte proliferation, survival and function during immune responses and homeostasis. Tax has been shown to transactivate a number of the common γ chain family of cytokines and the receptors, such as IL-2/IL-2R, IL-9, IL-15/IL-15R, and IL-21/IL-21R (Cross et al., 1987; Siekevitz et al., 1987; Azimi et al., 1998; Mariner et al., 2001; Mizuguchi et al., 2009). One of the most striking features of the cellular immune response in HAM/TSP patients is the increased numbers of memory and/or effector CD8^+^ T cells and also HTLV-1 Tax-specific cytotoxic CD8^+^ T cells (Jacobson et al., 1990; Nagai et al., 2001a). Since both IL-2 and IL-15 induce the proliferation and increase the cytolytic activity of NK and CD8^+^ T cells, it has been suggested that IL-2/IL-2R and IL-15/IL-15R autocrine loop may contribute to the pathogenesis of HAM/TSP (Azimi et al., 1999). In particular, IL-15 is critical for the development of NK cells and antigen-specific memory CD8^+^ T cells and is well characterized for its role in maintaining memory pools of CD8^+^ T cells. In HAM/TSP patients, *IL-15* mRNA level is up-regulated in non-T cells isolated from HAM/TSP patients and DC treated with HTLV-1 Tax protein *in vitro* (Azimi et al., 1999; Ahuja et al., 2006). IL-15 expression was also rapidly enhanced on the surface of CD14^+^ cells in HAM/TSP patients after the PBMC culture, more than those in ACs (Enose-Akahata et al., 2008). In addition, high expression of IL-15Rα has been reported in HTLV-1 Tax-specific CD8^+^ T cells, compared with CMV pp65-specific CD8^+^ T cells (Azimi et al., 2001). IL-15 stimulated HTLV-1 Tax-specific CD8^+^ T cells, but not CMV pp65-specific CD8^+^ T cells, to induce degranulation and IFN-γ expression (Enose-Akahata et al., 2008). Thus, the increase of the common g chain family of cytokines and receptors in HAM/TSP patients may be involved in increased proliferation and enhanced cytolytic activity and inflammatory cytokine production of HTLV-1-specific CD8^+^ T cells.

Increased levels of cytokines and the receptors driven by Tax further lead to upregulated protein expression and activated signaling cascades, such as JAK/STAT (Waldmann, 2015). IL-2/IL-15 binding to the β/γ chain complex results in heterodimerization of their cytoplasmic domains with activation of the Janus family tyrosine kinases, JAK1 and JAK3 (in association with β and γ chain, respectively). Activated JAK1 and JAK3 then phosphorylate signal transducer and activator of transcription proteins STAT3 and STAT5, respectively, to mediate IL-2 and IL-15 effects in immune cells (Waldmann, 2015). HAM/TSP patients showed increased STAT5 phosphorylation in T cells, which was inhibited by the blockade of IL-2Rα and IL-2/IL-15Rβ (Oh et al., 2011). These reports demonstrated that continuous stimulation driven through HTLV-1 Tax may be associated with the pathogenesis of HAM/TSP.

### The Blood–Brain Barrier (BBB)

The CNS is normally protected from infectious agents by a physiological structure called the blood–brain barrier (BBB), which consists primarily of a continuous endothelium with tight junctions. HAM/TSP develops upon infiltration of HTLV-1-infected lymphocytes into the CNS, mostly within the thoracic spinal cord. The tight junctions of the BBB endothelium in HAM/TSP patients are locally disorganized (Afonso et al., 2007, 2008). Matrix metalloproteinase (MMP) is known as a proteolytic enzyme, which is involved in degradation of many different components of extracellular matrix and is critical role in migration of leukocytes and damage of the BBB. It has been reported that high level of MMP-2 and MMP-9 was detected in the CSF and in infiltrating mononuclear cells of HAM/TSP patients, suggesting that MMP-2 and MMP-9 may cause disruption of BBB in HAM/TSP patients (Umehara et al., 1998). Later, MMP-9, but not MMP-2, was reported to be transactivated by Tax in HTLV-1-infected T cells (Mori et al., 2002). HTLV-1 infection has been also detected in astrocytes which interact with endothelial cells to form the BBB and may potentially also function as antigen-presenting cells (Lehky et al., 1995; Mendez et al., 1997). The expression of Tax in primary human astrocytomas and oligodendrogliomas resulted in robust induction of IL-1α, IL-1β, TNF-α, TNF-β, and IL-6 expression (Banerjee et al., 2007). These results suggested that increased inflammatory responses may cause disruption of BBB and the alteration of the BBB integrity may allow T cells to transmigrate into the CNS, resulting in neuroinflammation of HTLV-1-infected subjects.

Recruitment and extravasation of T cells through the BBB are favored by adhesion molecule-mediated interactions of circulating T cells with endothelial cells. Tax has been demonstrated to regulate cell adhesion molecules, such as intercellular adhesion molecule-1 (ICAM-1), vascular cell adhesion molecule 1 (VCAM-1) and cell adhesion molecule 1 (CADM1/TSLC1) (Valentin et al., 1997; Nejmeddine et al., 2009; Manivannan et al., 2016). Activated leukocyte cell adhesion molecule (ALCAM/CD166), a member of the immunoglobulin superfamily, is overexpressed on the surface of HTLV-1-infected lymphocytes, both in chronically infected cell lines and in primary CD4^+^CD25^+^ T cells from HAM/TSP patients (Curis et al., 2016). ALCAM expression was enhanced by Tax through the activation of the canonical NF-κB pathway (Curis et al., 2016). These results demonstrated that increase of ALCAM expression might facilitate the migration of HTLV-1-infected lymphocytes across the BBB endothelium.

### Tax in CSF of HAM/TSP Patients

Although *tax* mRNA and Tax protein are rarely or undetectable directly in fresh uncultured PBMCs of HAM/TSP patients, it has been reported that HTLV-1 *tax* mRNA was detected in cells of spinal cord and cerebellar sections, and HTLV-1 Tax protein could be also detected in CSF cells of HAM/TSP patients (Lehky et al., 1995; Moritoyo et al., 1999; Cartier and Ramirez, 2005). The increased expression of HTLV-1 Tax protein in the CSF cells was more frequent in HAM/TSP patients with shorter duration of illness (Cartier and Ramirez, 2005). The presence of Tax protein in CSF might cause direct cell damage and loss in the CNS or immune cells to activate and generate Tax-specific immune responses in HAM/TSP patients. Axonal degeneration in HAM/TSP patients occurs without HTLV-1 infection of neurons, suggesting that secreted Tax protein might be involved. Tax from culture media of MT-2 and PBMCs of HAM/TSP patients caused retraction of differentiated human neuroblastoma cells (Maldonado et al., 2011; Medina et al., 2014). Tax expression also sensitized primary astrocytomas to apoptosis (Banerjee et al., 2007). These observations suggest that the chronic Tax secretion from infected cells could be sufficient for producing a neurotoxic effect on the long axons of corticospinal tracts involved in progressive neurological disease. Recent increasing evidences revealed that extracellular vesicles called exosomes play critical roles in viral pathogenesis and control of host immune responses to viral infection that deliver these microvesicles that contain host and viral components, including proteins, mRNA, and microRNA (Anderson et al., 2016). HTLV-1 has been shown to incorporate viral products into shed exosomes. Jaworski et al. (2014) found exosomes derived from HTLV-1-infected cells to contain Tax protein and proinflammatory mediators as well as viral mRNA transcripts, including Tax, HBZ, and Env. Once viral proteins or viral mRNAs enter or are released from exosomes in the CNS, these viral products might be able to stimulate or damage resident cells in the CNS or sensitize uninfected target cells for lysis by HTLV-1-specific CD8^+^ T cells. These new findings suggest that incorporation of viral proteins and mRNAs into exosomes or alteration of host contents of immune cell derived exosomes may represent a mechanism by which viral antigens could be transported to the CNS and be associated with axonal degeneration and virus-specific immune responses in HAM/TSP.

## Molecular Pathogenesis of HBZ in HAM/TSP

*HBZ* encoded by the minus strand of the HTLV-1 proviral genome has been identified (Gaudray et al., 2002). While about 60% of ATL patients do not express the *tax* gene transcript in freshly isolated leukemic cells (Takeda et al., 2004), *HBZ* mRNA is ubiquitously expressed in HTLV-1-infected cells, ATL cells and PBMC of HTLV-1-infected individuals and promotes the growth and survival of the leukemic cells (Satou et al., 2006; Usui et al., 2008). HBZ interacts with CREB/ATF pathway, suppress Tax-mediated viral transcription, and selectively inhibits the classical NF-κB pathway (Matsuoka and Jeang, 2011). Previous *in vivo* studies also demonstrated that HBZ expression enhanced HTLV-1 infectivity, T cell proliferation and lymphoma (Arnold et al., 2006, 2008; Satou et al., 2011). While it has been reported that HBZ plays a crucial role in HTLV-1 infectivity and the generation and maintenance of the oncogenic process, there is less information about the role of HBZ on the molecular and cellular mechanisms leading to HAM/TSP.

### Localization of HBZ

The expression of *HBZ* mRNA was detected in PBMCs of HAM/TSP patients, which was significantly lower than in ATL patients but higher than in AC (Saito et al., 2009). Intriguingly, *HBZ* mRNA expression was correlated with PVL and disease severity in HAM/TSP patients (Saito et al., 2009). It remains unclear how expression of Tax (the plus strand transcription is minimized) and HBZ (constitutively expressed from the minus strand) is regulated in HTLV-1-infected cells, but there is increasing evidence that HBZ expression is detected in a different pattern or subset of CD4^+^ T cells from Tax-expressing CD4^+^ T cells. It has been demonstrated that high frequency of CD39^+^CD4^+^ T cells regardless of CD25 expression were detected in HAM/TSP patients compared to AC (Leal et al., 2013). When Tax and HBZ expression was examined in CD4^+^ T cells expressing CD25 and CD39 of HAM/TSP patients, *HBZ* mRNA expression was significantly correlated with CD39^+^CD4^+^ T cells while Tax expression was restricted to CD25-expressing CD4^+^ T cells regardless of CD39 expression (Leal et al., 2013). Moreover, a recent study using single molecule RNA fluorescent *in situ* hybridization targeted to the transcripts of *tax* or *HBZ* genes revealed that *tax* mRNA expression was enhanced in the absence of *HBZ* mRNA in patient-derived HTLV-1^+^ T cell clones while *HBZ* mRNA expression was increased in cells with high *tax* mRNA expression (Billman et al., 2017). In addition, it has been demonstrated that at the single cell level, *HBZ* mRNA was expressed at much lower level, detected in more uniformly across cells compared to *tax* mRNA, and was not expressed in all T cell clones (Billman et al., 2017). These studies suggested that HBZ expression might be compartmentalized or cooperate with Tax expression in HTLV-1-infected T cells. A recent study demonstrated that CTCF, a zinc-finger protein and a key regulator of chromatin structure and function, bound to HTLV-1 and formed loops between proviral and host genes to regulate HTLV-1 proviral transcription and RNA splicing (Satou et al., 2016). Further molecular virological studies would be necessary to understand how the pattern of proviral transcription and latency is regulated in HTLV-1-infected individuals.

Unlike *HBZ* mRNA, HBZ protein was rarely detected in HAM/TSP patients (Shiohama et al., 2016). Previously, higher nuclear retention of *HBZ* mRNA and localization of HBZ in the nucleus have been demonstrated (Hivin et al., 2005; Rende et al., 2011), suggesting that HTLV-1 might favor viral persistence by reducing HBZ translation to escape the infected cells from HBZ-specific immune responses. However, HBZ protein has been recently reported to be localized in the cytoplasm of T cells depending on the expression of THEMIS (thymocyte-expressed molecule involved in selection), a recently identified T lineage-restricted protein (Kinosada et al., 2017). HBZ interfered complex formation of THEMIS with Grb2 and SHP-2, which resulted in inhibition of suppressive function of co-inhibitory receptors, such as T cell immunoglobulin and ITIM domain (TIGIT) and program death-1 (PD-1), and enhanced activation of T cells (Kinosada et al., 2017). Localization of HBZ protein was also reported in the cytoplasm of PBMCs of HAM/TSP patients (Baratella et al., 2017). Interestingly, HBZ protein is almost exclusively in CD4^+^ T cells irrespective of co-expression of CD25 (Baratella et al., 2017). Thus, HBZ expression may be compartmentalized or cooperate with Tax expression in HTLV-1-infected CD4^+^ T cells of HAM/TSP patients and may allow the virus to evade the host immune system. These findings of HBZ might shed light on a new molecular basis for a role of HBZ in the pathogenesis of HAM/TSP.

### Inflammation by HBZ

As HBZ closely cooperates with Tax in many molecular mechanisms (Matsuoka and Jeang, 2011), opposing functions between HBZ and Tax have been also demonstrated in both *in vitro* and *in vivo* studies. HBZ enhanced TGF-β signaling and FoxP3 expression to induce Tregs from naïve CD4^+^ T cells while Tax reduced TGF-β signaling and FoxP3 expression in CD4^+^ T cells (Grant et al., 2008; Zhao et al., 2011). HBZ suppresses the *IFN*-γ gene transcription through inhibition of AP-1 and NFAT while Tax activates the *IFN*-γ gene promoter (Sugata et al., 2012). Interestingly, HBZ transgenic (HBZ-Tg) mice develop both T-cell lymphomas and chronic inflammation in lung and skin (Satou et al., 2011). Currently, there is no report that HBZ-Tg mice develop inflammatory neurologic diseases and it remains unknown how tissue specificity of HTLV-1-associated inflammatory diseases is determined, but similar immunological features with HAM/TSP patients have been demonstrated in HBZ-Tg mice. HBZ-Tg mice has been reported to show the increased effector/memory CD4^+^ T cells while effector/memory CD4^+^ T cells with high Tax-expression have been exhibited in HAM/TSP patients (Hanon et al., 2001; Satou et al., 2011). Further studies have demonstrated that Tregs of HBZ-Tg mice tend to lose expression of FoxP3 and Helios, leading to increased IFN-γ-expressing proinflammatory cells, associated with enhanced cell adhesion and migration of CD4^+^ T cells of HBZ-Tg mice (Yamamoto-Taguchi et al., 2013). Importantly, decreased Helios expression and enhanced cell adhesion molecules observed in HBZ-Tg mice were also detected in CD4^+^ T cells of HAM/TSP patients (Yamamoto-Taguchi et al., 2013). In addition, the conserved non-coding sequence 2 region of the *Foxp3* gene was hypermethylated in Tregs of HBZ-Tg mice, which is a characteristic of induced Tregs (Yamamoto-Taguchi et al., 2013). HAM/TSP patients also showed decreased demethylation of the *Foxp3* gene in CD4^+^CD25^+^ T cells, compared to NDs, which correlated with the decreased suppressive capacity of CD4^+^CD25^+^ T cells in HAM/TSP patients (Anderson et al., 2014). These results suggested that HBZ may be able to convert into the proinflammatory phenotype of HBZ-expressing T cells, suggesting that HBZ plays an important role in the disease process of HAM/TSP.

## Immune Response Against Tax and HBZ in HAM/TSP

### Tax-Specific Immune Responses

Tax is an immunodominant antigen recognized by HTLV-1-specific cytotoxic CD8^+^ T cells (CTLs) (Jacobson et al., 1990). CD8^+^ T cells play a crucial role in immunity against HTLV-1 through their ability to secrete various factors that suppress viral replication and kill infected target cells in HTLV-1-infected subjects (Hanon et al., 2000b; Vine et al., 2004). However, in HAM/TSP patients, the frequency of HTLV-1 Tax-specific CD8^+^ T cells were even higher in CSF than in peripheral blood and were correlated with HTLV-1 PVL (Greten et al., 1998; Kubota et al., 1998; Nagai et al., 2001b). A recent study demonstrated that a more atrophic spinal cord in HAM/TSP was associated with higher percentage of inflammatory CD8^+^ T cells and HTLV-1 PVL in CSF of HAM/TSP patients (Azodi et al., 2017). Moreover, it has been demonstrated that HTLV-1 Tax-specific CD8^+^ T cells as well as CD4^+^ T cells expressing HTLV-1 proteins were detected in the parenchyma of the spinal cords, suggesting that the interaction between HTLV-1-specific CTLs and HTLV-1-infected CD4^+^ T cells may cause bystander damages to resident cells in the CNS (Matsuura et al., 2015).

While virus-specific antibodies play an important role in the control of viral infections in the CNS, intrathecal antibody synthesis appear to be associated with both protective and pathogenic function in chronic infection and immune-mediated diseases of the CNS. Intrathecal antibody synthesis against HTLV-1 has been also confirmed by the presence of HTLV-1-specific antibodies and oligoclonal IgG band in CSF of HAM/TSP patients (Gessain et al., 1988; Grimaldi et al., 1988; Link et al., 1989). Intrathecal antibody response to HTLV-1 inversely correlates with higher PVL and a worse prognostic outcome (Puccioni-Sohler et al., 1999). Moreover, antibodies against two HTLV-1 viral products, Tax and Gag p24, have been reported to cross-react with host antigens, heterogeneous ribonucleoprotein A1 (hnRNP A1) and peroxiredoxin-1 (PrX-1), respectively, suggesting that molecular mimicry may play a role in the pathogenesis of HAM/TSP, suggesting a role for molecular mimicry between an infectious agent and the CNS (Levin et al., 2002; Lee et al., 2008). Therefore, HTLV-1 Tax specific immune responses might be immunopathogenic, rather than protective, in HAM/TSP patients, due to high cytotoxicity, the production of inflammatory cytokines such as IFN-γ and TNF-α, associate with damage to the CNS.

### HBZ-Specific Immune Responses

HBZ is also an immunogenic protein recognized by HBZ-specific CTL clones (Suemori et al., 2009; Macnamara et al., 2010). HBZ-specific CD8^+^ T cells are detected in AC and HAM/TSP patients, and HBZ-specific CTL clones were able to lyse naturally infected cells isolated from AC and HAM/TSP patients, but not ATL patients (Suemori et al., 2009; Macnamara et al., 2010). However, the binding affinity of HBZ peptides to HLA class I molecules was found to be significantly weaker than that of peptides from Tax, and the frequency of HBZ-specific CD8^+^ T cells was very low in peripheral blood (Macnamara et al., 2010; Hilburn et al., 2011).

Antibody response against HBZ was detected in serum/plasma of HTLV-1-infected subjects, but the frequency of the subjects with anti-HBZ antibody was about 10–16% in ACs, ATL, and HAM/TSP patients and did not discriminate between clinical status (Enose-Akahata et al., 2013; Shiohama et al., 2016). In addition, antibody responses against HBZ was detectable in the CSF of HAM/TSP patients, but was not dramatically elevated, suggesting that HBZ-specific antibody is not intrathecally synthesized (Enose-Akahata et al., 2013).

It has been suggested that the low frequency and affinity of HBZ-specific immune responses may be consequence of the low expression and antigenicity of HBZ in infected cells. Unlike Tax-specific immune responses, it might be difficult to induce effective HBZ-specific immune responses in HAM/TSP patients although the *HBZ* gene is constantly expressed while the *tax* gene is sporadically transcribed. Thus, HTLV-1-infected cells might be able to escape from host immune responses for long periods and result in persistence of HTLV-1 in infected individuals.

## Conclusion

Both Tax and HBZ play critical roles in immune dysregulation in HAM/TSP. However, it is still unknown how expression of Tax and HBZ are regulated and how Tax and HBZ cooperate in naturally infected cells. Therapies that control the expression of HTLV-1 gene products might be effective in preventing the development of HAM/TSP. In addition, effective regulation or induction of HTLV-1 specific immune responses might improve the prognosis of patients with this disorder. Further studies will be necessary to identify the mechanism by which HTLV-1 gene products or other factors including HTLV-1-specific immune responses contribute to the pathogenesis of HAM/TSP.

## Author Contributions

YE-A contributed to paper writing. AV contributed to discussion. SJ supervised and contributed to discussion and writing.

## Conflict of Interest Statement

The authors declare that the research was conducted in the absence of any commercial or financial relationships that could be construed as a potential conflict of interest.
